# ﻿*Barbodesklapanunggalensis*, a new species of blind subterranean fish (Cypriniformes, Cyprinidae) from Klapanunggal karst area, West Java, Indonesia, with notes on its conservation

**DOI:** 10.3897/zookeys.1229.135950

**Published:** 2025-02-24

**Authors:** Kunto Wibowo, M. Iqbal Willyanto, Anik Budhi Dharmayanthi, Cahyo Rahmadi, Daniel Natanael Lumbantobing

**Affiliations:** 1 Museum Zoologicum Bogoriense, Research Center for Biosystematics and Evolution, National Research and Innovation Agency, Cibinong 16911, Jawa Barat, Indonesia Research Center for Biosystematics and Evolution, National Research and Innovation Agency Cibinong Indonesia; 2 Indonesian Speleological Society, Bogor 16121, Jawa Barat, Indonesia Indonesian Speleological Society Bogor Indonesia; 3 Species Obscura, Depok 16434, Jawa Barat, Indonesia Species Obscura Depok Indonesia

**Keywords:** Cave-dwelling fish, coloration, endemic, morphology, silvery barbs

## Abstract

*Barbodesklapanunggalensis***sp. nov.** is described on the basis of two specimens collected from the subterranean cave system of Klapanunggal karst area, Bogor Regency, West Java Province, Java Island, Indonesia. The new species is unique among its congeners in completely lacking eyes, its ocular vestige being marked by an orbital concavity fully covered with skin, and with no orbital rim. It also differs distinctly from most species of *Barbodes* by having relatively long paired fins (pectoral fin 26.0–31.4% SL; pelvic fin 21.5–24.4% SL), the adpressed tips of each overlapping the basal area of the adjacent posterior (pelvic and anal, respectively) fins; and the presence of a short pelvic axillary scale with a rounded posterior margin. *Barbodesklapanunggalensis***sp. nov.** is further distinguished from all congeners by the following combination of characters: head length 32.9–35.3% SL; pre-pectoral fin length 32.6–33.6% SL; pre-pelvic fin length 54.0–59.6% SL; anal-fin base length 9.7–11.8% SL; caudal peduncle depth 13.2–18.2% SL; completely nonpigmented body; and all fins with translucent interradial membranes and light cream to brownish rays. As reflected by its highly troglomorphic features, the new species is strictly adapted to cave habitats. Its small population size, coupled with a high level of potential threats to its known habitat, suggest that future conservation efforts will become necessary.

## ﻿Introduction

The cyprinid fish genus *Barbodes* Bleeker, 1859 is a group of small to medium-sized silvery barbs comprising hitherto 44 benthopelagic species known from inland freshwater bodies in Southeast Asia and southern China (Fricke et al. 2024). Almost two-thirds of the species diversity of *Barbodes* resides within the Philippines archipelago, as it harbors 28 species, all inferred to belong to the *B.binotatus* species group (Kottelat 2013). The remaining non-Philippines *Barbodes* (16 species) live throughout Sundaland, Indochina, and southern China. In Sundaland, two species among the first *Barbodes* known to science—*Barbodesbinotatus* (Valenciennes in Cuvier & Valenciennes, 1842) *sensu stricto* and *Barbodesmicrops* (Günther, 1868)—were originally described from the Indonesian island of Java (Roberts 1989; Kottelat 2013; Kottelat and Lim 2021; Tan and Husana 2021; Fricke et al. 2024).

The taxonomy of *Barbodes* has a convoluted history, largely owing to its vague original description, accompanied by ambiguous generic diagnoses for several assemblages of Asian barbs as defined in earlier literature. Most members of the genus *Barbodes*, as currently known, were previously classified in the catch-all genus *Puntiussensu lato*, together with a variety of other relatively small-bodied Asian barb lineages, as recognized by most authors at that time (e.g., Kottelat 1989; Kottelat et al. 1993). At the same time, the name *Barbodes* in Southeast Asia had been assigned to another lineage containing fewer species of larger-bodied barbs (see also Rainboth 1996), which has later been transferred entirely by Kottelat (1999) to the genus *Barbonymus* Kottelat, 1999 as recognized to date. Kottelat (1999) also treated *Barbodes* as the junior subjective synonym of *Systomus* McClelland, 1838 as defined earlier by Rainboth (1996). As it was limited to Cambodian Mekong, only a few species of *Puntiussensu lato* were included by Rainboth (1996) in the genus *Systomus* per his definition. Despite using the definition of Rainboth (1996) in synonymizing *Barbodes*, Kottelat (1999) disagreed with the partial use of *Systomus* by the former author given such a limited geographic area alongside unclear generic delineation. Thus, some subsequent works (e.g., Kottelat 2000; Kottelat 2001b) disregarded the genus *Systomussensu*Rainboth (1996) and used *Puntiussensu lato* for most species now considered *Barbodes*.

It took more than a decade after Kottelat (1999) for the generic name *Barbodes* to be revived when, in their revision of South Asian *Puntiussensu lato*, Pethiyagoda et al. (2012) pointed out the availability of *Barbodes* for an assemblage of Southeast Asian barbs; this was followed by Kottelat (2013), who complementarily reviewed the Southeast Asian counterparts of this catch-all genus. In this review, Kottelat (2013) splitted the members of *Puntiussensu lato* from Southeast Asia into eight genera: four genera being given earlier available names (i.e., *Puntius* Hamilton, 1822; *Barbodes*; *Systomus*; and *Pethia* Pethiyagoda, Meegaskumbura & Maduwage, 2012), whereas four other ones described as new genera (i.e., *Desmopuntius*, *Oliotius*, *Puntigrus*, and *Striuntius*). In this review, Kottelat (2013) classified 41 valid species from Southeast Asia in the genus *Barbodes*. Subsequently, Zhang and Zhao in Fricke et al. (2024) reported the validity of the Chinese species *Barbodespolylepis* Chen & Li, 1988. Based on molecular phylogenies in their study, Ren et al. (2020) transferred a species previously regarded by Kottelat (2013) as a valid *Barbodes*—formerly *B.lateristriga* (Valenciennes, 1842)—into the genus *Striuntius*. More recently, Tan and Husana (2021) described a new cave-dwelling species—*Barbodespyrpholeos*—from the Philippines, while Kottelat and Lim (2021) described two new species—*Barbodessellifer* and *Barbodeszakariaismaili*—from Malay Peninsula, Sumatra, and neighboring islands (Riau archipelago and Singapore).

As provided by Kottelat (2013), *Barbodes* is readily distinguished from other genera in the family Cyprinidae in having the following color pattern: juveniles with 3–5 black dots along the body mid-lateral surface, including a single dot in the middle of the caudal-fin base and below the dorsal-fin origin; larger individuals with the mid-lateral dots usually multiplied to form a stripe, while the spot below the dorsal-fin origin often becoming a large blotch (see also Pethiyagoda et al. 2012). The genus is also characterized by a combination of the following morphological features: the posterior margin of the last simple dorsal-fin ray is serrated; the presence of maxillary barbels; both lips are smooth and thin with the postlabial groove being interrupted medially; a total of 22–32 scales along the lateral-line series with a complete or incomplete perforation; the formula for the transverse scale rows between the dorsal-fin origin and the ventral midline anterior to the pelvic-fin insertion is ½4/1/4½; 12 circumpeduncular scale rows; and 12–15 gill rakers on the first gill arch (Kottelat 2013).

**Figure 1.**
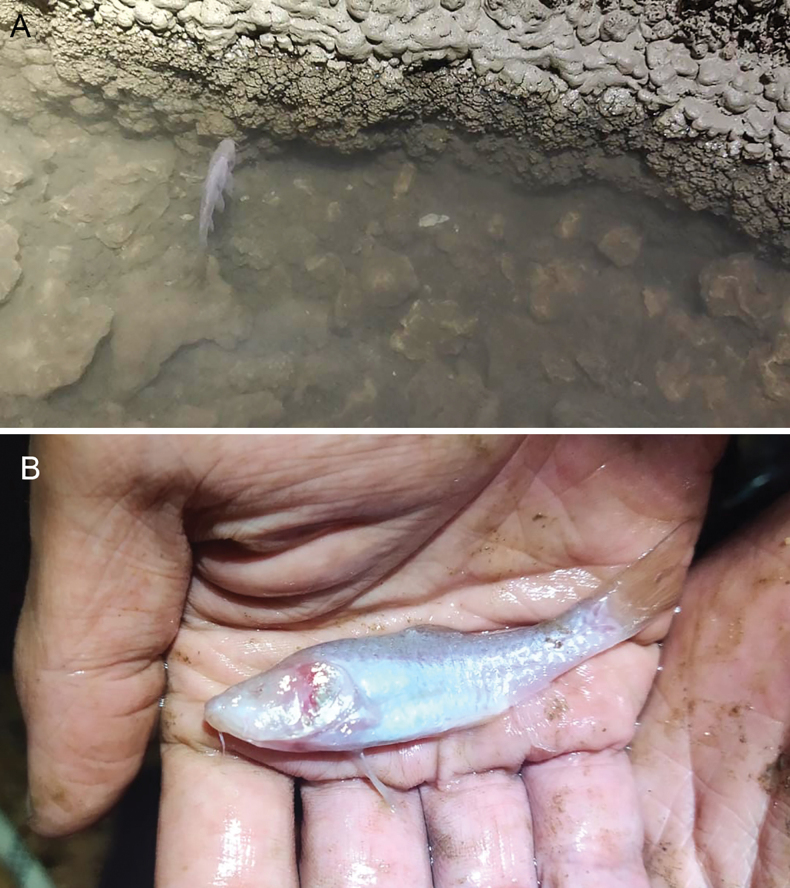
Photographs of the holotype of *Barbodesklapanunggalensis* sp. nov. **A** in situ photograph, from video taken in 2020 by MIW **B** picture taken in 2020 by MIW.

**Figure 2.**
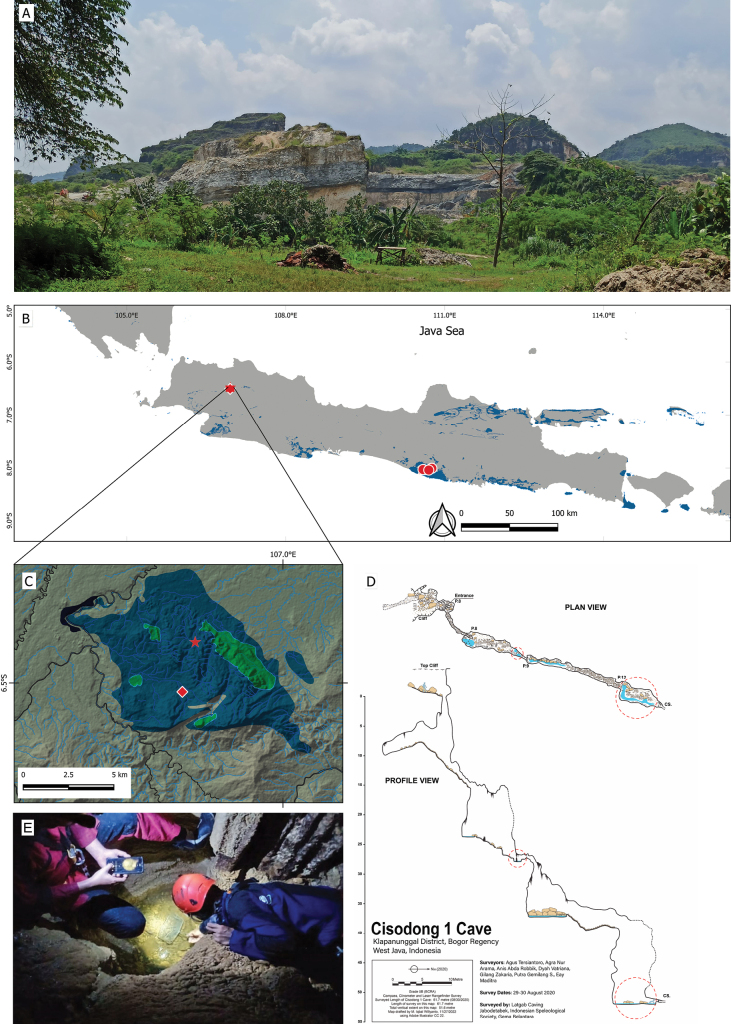
Distribution maps and habitat of *Barbodes* species **A** landscape of Klapanunggal karst area **B, C** distributions of *B.klapanunggalensis* sp. nov. (star), *B.microps* (circles), and B.cf.microps (diamond) on Java Island **C** Klapanunggal karst area, blue and green areas indicate limestone formation and protected karst area, respectively **D** plan and profile views of Cisodong 1 Cave in Klapanunggal karst area, small and large red circles indicate type locality and a second location, respectively, of *B.klapanunggalensis* sp. nov. **E** habitat of *B.klapanunggalensis* sp. nov.

Two blind specimens of a cyprinid fish (Fig. 1) collected during an exploration of the subterranean cave system, Klapanunggal karst area in Bogor Regency, West Java Province, Indonesia (Fig. 2), were characterized by a uniformly silvery-whitish body (fresh coloration) entirely lacking melanophore pigmentation. Despite the absence of pigmented markings, all other morphological characters agreed well with the non-pigment diagnostics of the genus *Barbodes*, given by Kottelat (2013). In being nonpigmented, the specimens were most similar to two stygobitic *Barbodes* species, *B.microps* (Java) and *B.pyrpholeos* (the Philippines). However, they differed from both aforementioned species and also the congeneric epigean Javan species *B.binotatus* in several distinct morphological characters, and thus described herein as a new species of *Barbodes*.

## ﻿Materials and methods

Counts and measurements in general followed Kottelat (2001a), Kottelat and Freyhof (2007), and Kottelat and Lim (2021), except for the following counts and measurements: transverse scales above the lateral line were counted from the scale at the origin of the dorsal fin downward to the scale just before the lateral-line scale; transverse (1^st^) scales below the lateral line were counted from the scale at the origin of the pelvic fin upward to the scale before the lateral-line scale; transverse (2^nd^) scales below the lateral line were counted from the scale at the origin of the anal fin upward to the scale before the lateral-line scale; dorsal head length was measured from the anteriormost point of the snout to the anterior margin of the anteriormost predorsal scale; head depth was measured at the anterior margin of the anteriormost predorsal scale; pre-pectoral fin length was measured from the anteriormost point of the snout to the anterodorsalmost part of the pectoral fin insertion. The vertebral number was counted from radiographs, and the last two soft rays of the dorsal and anal fins as 1½, each pair being associated with a single pterygiophore. Standard and head lengths are expressed as SL and HL, respectively. Curatorial procedures followed Motomura and Ishikawa (2013). The specimens examined in this study are held in the Natural History Museum, England (**BMNH**; London) and the Museum Zoologicum Bogoriense, Indonesia (**MZB**; Bogor).

### ﻿Comparative material examined

All specimens were collected from Indonesia. *Barbodesmicrops*: • BMNH 1845.6.22.334–336, syntypes of *Barbusmicrops*, 103.4 mm SL, 93.6 mm SL, 77.8 mm SL, Java • BMNH 1845.422.341–342, syntypes of *Barbusmicrops*, 42.2 mm SL, 43.8 mm SL, Java. Barbodescf.microps: • MZB.60, 14 specimens, 48.6–107.5 mm SL [10 previously examined by Haryono (2006); all with functional eyes], Sileuwi Cave, Klapanunggal karst area, Loeloet Village, Klapanunggal District, Bogor Regency, West Java Province. *Barbodesbinotatus*: • MZB.16585, 3 of 7 specimens examined, 46.4–51.2 mm SL, Suci River, Pacarejo, Semanu, Gunungkidul, Yogyakarta, D. Wowor and Mulyadi, 4 Aug. 2006 • MZB.17723, 4 of 7 specimens examined, 48.6–58.1 mm SL, Kiskendo Cave, Purworejo, Central Java, R. Hadiaty and M. Wahyudin, 27 Jun. 2009 • MZB.17843, 6 of 7 specimens examined, 57.0–78.7 mm SL, Ciliwung River, Bogor Botanical Garden, Bogor, West Java, 06°35'37.9"S, 106°48'01.4"E, R. Hadiaty et al., 1 Aug. 2009 • MZB.22909, 77.1 mm SL, Teneran River, Kali Gono, Kali Gesing, Purworejo, Central Java, R. Hadiaty et al., 25 May 2015 • MZB. 22914, 5 specimens, 50.7–61.4 mm SL, Kotak River, Tlogoguwo, Kali Gesing, Purworejo, Central Java, R. Hadiaty et al., 26 May 2015 • MZB.22917, 2 of 11 specimens examined, 54.5–54.9 mm SL, Gesing River, Kali Gono, Kali Gesing, Purworejo, Central Java, R. Hadiaty et al., 27 May 2015.

## ﻿Results

### ﻿ Barbodes klapanunggalensis

Taxon classificationAnimaliaCypriniformesCyprinidae

Wibowo, Rahmadi & Lumbantobing sp. nov.

496E3FA0-FAC9-534C-A265-2A80715B7F91

https://zoobank.org/8C83CE9C-D3FC-47C0-82FA-A1AFC0D57D6A

**Figure 3.**
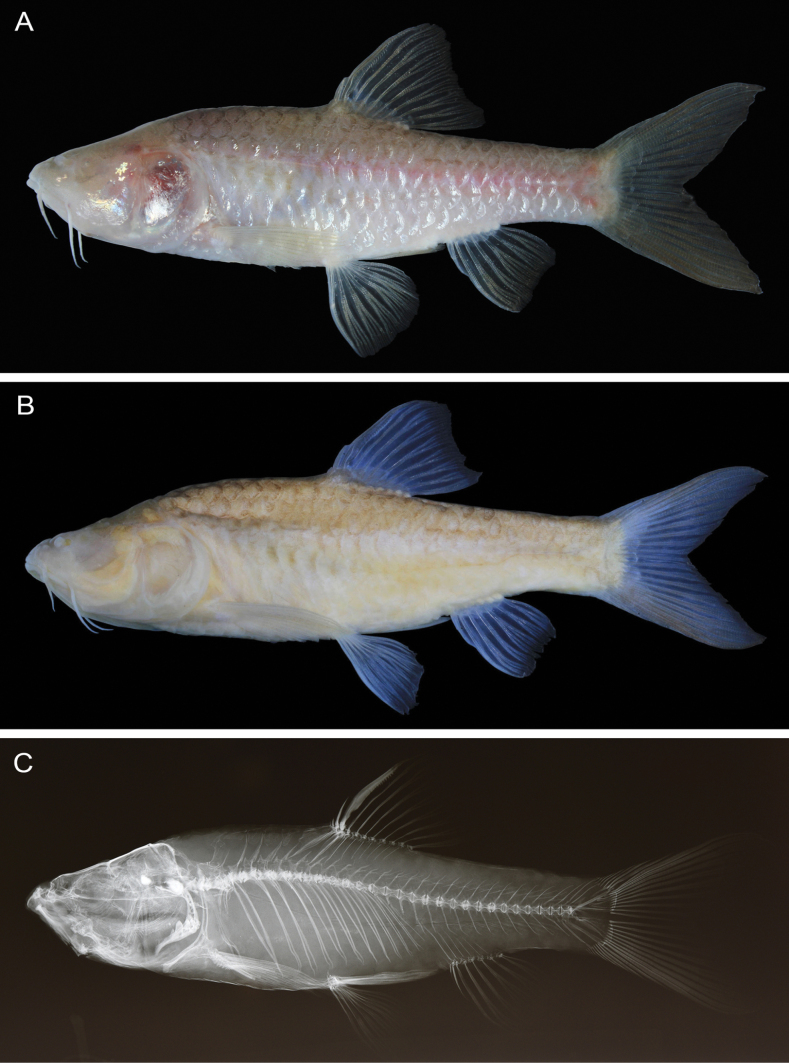
*Barbodesklapanunggalensis* sp. nov., MZB.26657, holotype, 63.8 mm SL **A** fresh **B** preserved **C** radiograph.

**Figure 4.**
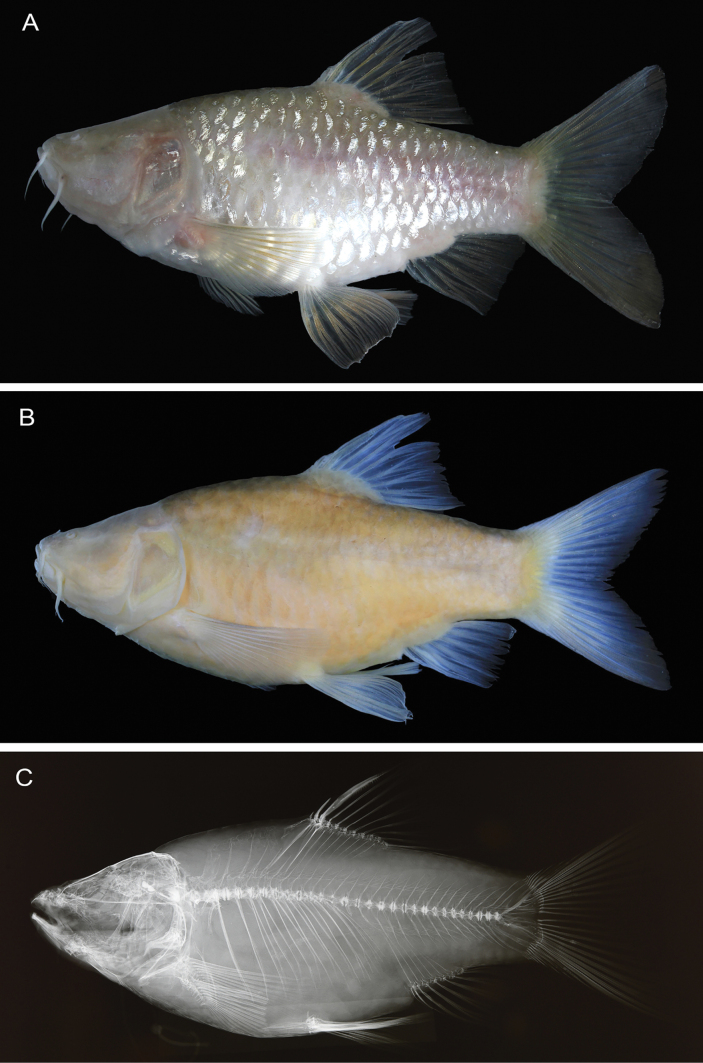
*Barbodesklapanunggalensis* sp. nov., MZB.26656, paratype, 73.0 mm SL **A** fresh **B** preserved **C** radiograph.

**Figure 5.**
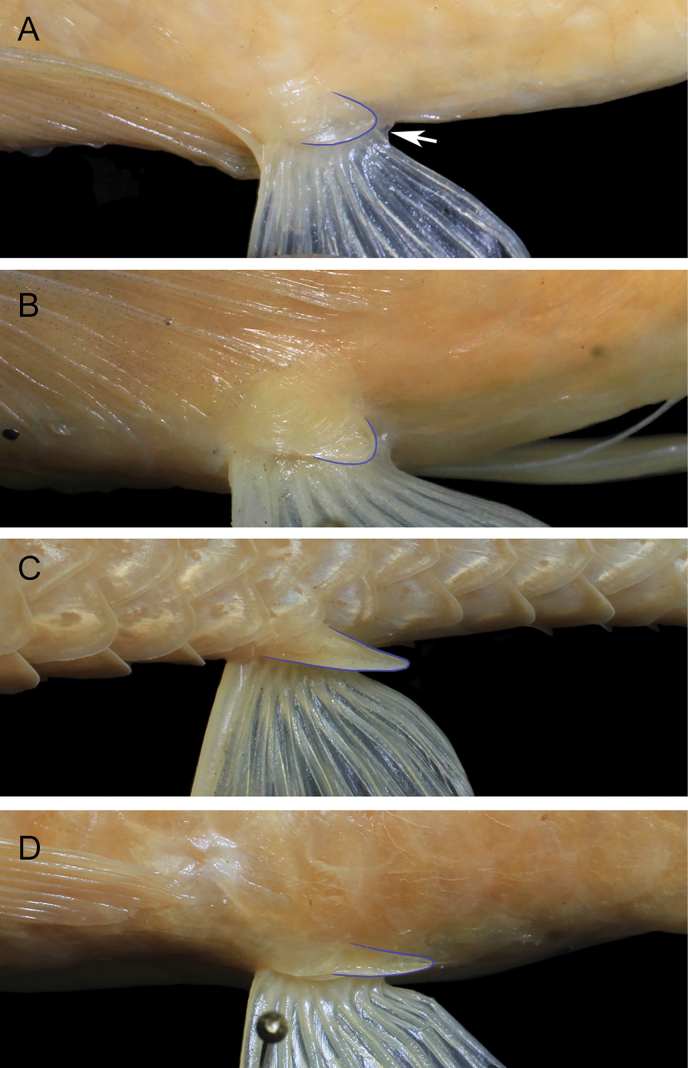
Pelvic fin axillary scale in **A, B***Barbodesklapanunggalensis* sp. nov., MZB.26657, holotype, 63.8 mm SL, MZB.26656, paratype, 73.0 mm SL, respectively **C***Barbodesbinotatus*, MZB.17843, 78.7 mm SL **D**Barbodescf.microps, MZB.60, 89.8 mm SL. Arrow in **A** indicates posterior end of pelvic fin base membrane.

**Figure 6.**
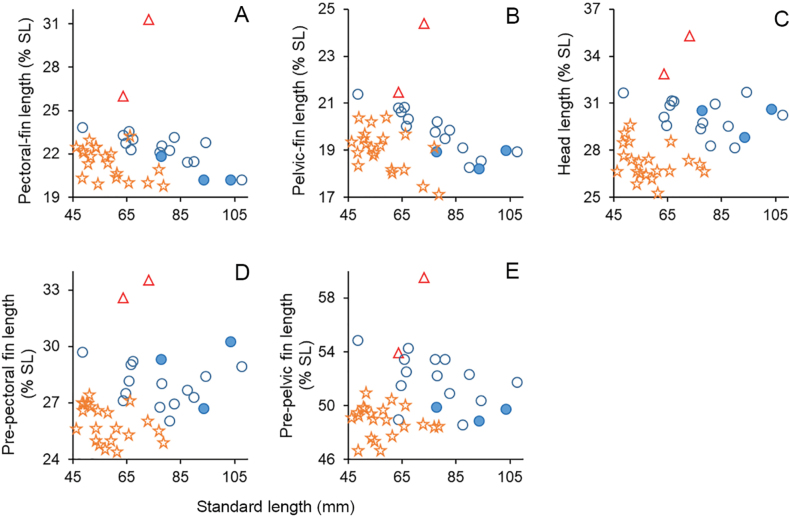
Relationships of **A** pectoral fin **B** pelvic fin **C** head **D** pre-pectoral fin, and **E** pre-pelvic fin lengths (all as % SL) to SL in *Barbodesklapanunggalensis* sp. nov. (triangles), *Barbodesbinotatus* (stars), *Barbodesmicrops* (closed circles), and Barbodescf.microps (opened circles).

Figs 1 , 3 , 4 , 5 , 6 , Table 1 New English name: Klapanunggal Blind Cave Barb New Indonesian name: Wader Gua Buta Klapanunggal

#### Type materials.

***Holotype*. Indonesia** • 63.8 mm SL; Java Island, West Java Province, Bogor Regency, Klapanunggal District, Nambo Village, Klapanunggal karst area, subterranean cave system of Cisodong 1 Cave; altitude 212 m a. s. l.; 3 July 2022; M. I. Willyanto, M. Yusmaryudi, and A. Novriansyah legs.; hand net; MZB.26657.

***Paratype*.**MZB.26656, 73.0 mm SL, same data as holotype.

#### Diagnosis.

A species of *Barbodes* distinguished from all its congeners by the absence of eyes, as the eye is vestigially replaced by an orbital concavity being fully closed by an epidermal layer, while lacking the orbital rim. The species is also uniquely diagnosed by having relatively long pectoral and pelvic fins, with their adpressed tips each extending past the vertical through the insertion or origin of the next fin posterior to the tip, as to further pass posteriorly about two scales in anteroposterior; and by the presence of a relatively short and rounded axillary pelvic-fin scale, with tip not reaching posterior edge of pelvic-fin base. It is further distinguished from other *Barbodes* species in having by the following combination of characters: head length 32.9–35.3% of SL; pre-pectoral fin length 32.6–33.6% of SL; pre-pelvic fin length 54.0–59.6% of SL; pectoral fin length 26.0–31.4% of SL; pelvic fin length 21.5–24.4% of SL; anal-fin base length 9.7–11.8% of SL; caudal peduncle depth 13.2–18.2% of SL; body without pigmentation (black dots, bars, stripes, blotches, and triangular markings all absent from lateral surface); all fins with translucent interradial membrane and light cream to brownish rays.

#### Description.

Data for holotype presented first, followed by paratype data in parentheses (if different). Selected meristic and morphometric data given as percentages of SL in Table 1. General appearance shown in Figs 3A, B, 4A, B.

**Table 1.** Counts and measurements (expressed as percentages of standard length) of *Barbodesklapanunggalensis* sp. nov., *B.binotatus*, *B.microps*, and B.cf.microps.

	*B.klapanunggalensis* sp. nov.		*B.binotatus*	*B.microps*			B.cf.microps
	Holotype	Paratype	Non-types	Syntypes of *Barbusmicrops*			Non-types
	MZB.26657	MZB.26656	*n* = 21	BMNH 1845.6.22.334	BMNH 1845.6.22.335	BMNH 1845.6.22.336	*n* = 14
Standard length (mm)	63.8	73.0	46.4–78.7	103.4	93.6	77.8	48.6–107.5
Counts							
Dorsal-fin rays	iv, 8½	iii, 8½	iii–iv (iii), 8½–9½ (8½)	iii, 8½	iii, 8½	iii, 7½	iii, 8½
Anal-fin rays	iii, 5½	iii, 5½	iii, 5½	iii, 5½	iii, 5½	iii, 5½	iii, 5½
Pectoral-fin rays	15	14	14–16 (15)	15	15	15	13–16 (15)
Pelvic-fin rays	10	9	8–10 (9)	9	9	9	8–10 (9)
Principal caudal-fin rays	10+9	10+9	10+9	10+9	10+9	10+9	10+9
Pored lateral-line scales	24+1	22+1	22–26 (24)+0–2 (2)	24+2	24+2	24+2	22–25 (23 or 24) + 0–2 (2)
Pre-dorsal scales	10	10	9–12 (10 or 11)	10	11	11	10 or 11
Transverse scales above lateral line	4½	4½	4½–5½ (4½)	4½	4½	4½	4½
Transverse (1^st^) scales below lateral line	3½	3½	3–4½ (3½)	3½	3½	3½	3½
Transverse (2^nd^) scales below lateral line	3½	3½	3½–4½ (3½)	3½	3½	3½	3½
Circumpeduncular scales	12	12	12	12	12	12	12
Gill rakers	ca. 12	ca. 12	10–13 (12)	13	11	11	11–13 (12)
Vertebrae	29	28	–	29	29	30	–
Measurements							
Body depth	27.6	43.1	28.3–35.1 (31.0)	32.1	33.5	31.6	31.3–39.4 (36.3)
Body width	14.1	22.5	13.4–18.7 (15.9)	17.1	17.2	15.9	16.0–19.2 (17.6)
Head length	32.9	35.3	25.3–29.7 (27.3)	30.6	28.8	30.6	28.2–31.7 (30.2)
Dorsal head length	23.5	25.7	18.4–21.9 (20.1)	23.8	21.8	21.3	21.0–25.3 (22.4)
Head depth	21.4	27.6	19.8–23.3 (21.3)	22.3	21.1	21.3	21.8–24.1 (22.9)
Pre-dorsal fin length	53.0	56.6	51.5–56.5 (54.1)	52.6	57.0	54.8	54.8–61.7 (57.8)
Pre-pectoral fin length	32.6	33.6	24.5–27.5 (26.0)	30.2	26.7	29.3	26.0–29.7 (27.9)
Pre-anal fin length	73.6	77.2	69.7–74.7 (72.0)	72.6	72.1	72.3	72.0–79.3 (76.0)
Pre-pelvic fin length	54.0	59.6	46.7–51.0 (48.9)	49.7	48.9	49.9	48.6–54.9 (52.0)
Dorsal fin length	24.3	25.9	21.4–26.7 (23.9)	22.2	broken	broken	20.1–27.8 (24.2)
Dorsal-fin base length	15.1	16.4	14.1–17.2 (15.7)	15.1	14.9	15.9	14.3–17.4 (15.8)
Pectoral fin length	26.0	31.4	19.9–23.3 (21.5)	20.2	20.2	21.8	20.2–23.8 (22.5)
Pelvic fin length	21.5	24.4	17.2–20.5 (19.0)	19.0	18.2	18.9	18.3–21.4 (19.9)
Anal fin length	17.7	19.6	15.3–21.2 (17.6)	15.8	16.2	18.4	15.4–19.7 (17.4)
Anal-fin base length	9.7	11.8	9.1–11.2 (10.3)	8.7	10.0	9.1	8.9–12.1 (10.4)
Caudal peduncle depth	13.2	18.2	13.2–15.9 (14.7)	12.1	13.3	12.7	13.2–15.7 (14.4)
Caudal peduncle length	18.8	18.4	17.4–21.9 (20.0)	18.5	19.5	19.4	16.3–20.2 (18.0)

Body deep, laterally moderately compressed along anterior portion, progressively more compressed posteriorly. Greatest body depth at vertical through dorsal-fin origin. Dorsal profile of head posterodorsally slanted overall, with slight concavity along supraorbital profile. Limit between head and trunk marked by slight convexity of anterior predorsal profile. Dorsal profile of anterior body slightly arched anteriorly, nearly flat posteriorly (entirely posterodorsally slanted in paratype). Snout slightly rounded. Mouth subterminal, marked by two pairs of maxillary barbels, anterior pair shorter than posterior pair. Eye absent, ocular vestige marked by orbital concavity completely covered by skin, orbital rim absent. A short flap on upper posterior edge of low membranous tube associated with anterior nostril. Cycloid scales covering body, not extending onto fin rays or membranes, except basally on dorsal, anal, and caudal fins. Pelvic-fin axillary scale short, with rounded tip (slightly projecting on right side of holotype), not reaching vertical through posterior edge of pelvic-fin base (Fig. 5A, B).

Dorsal fin with distal profile slightly concave, 4 (3) unbranched spinous rays, 8½ soft rays. First unbranched dorsal-fin ray very small; second ray about one-quarter length of third; last unbranched ray wide at base, progressively narrowing distally, with 10 (19) serrae along posterior edge of stiffened upper half. All soft dorsal-fin rays branched, first branched ray longest, slightly longer than fourth (third) unbranched ray. Pectoral fin slightly rounded, with 15 (14) rays; anteriormost ray unbranched, its adpressed tip extending past vertical through pelvic fin insertion. Pelvic fin with 10 (9) rays; anteriormost ray unbranched, its adpressed tip just reaching (extending past) anal fin origin; pelvic fin insertion about level with dorsal fin origin. Anal fin with 3 unbranched and 5½ branched rays; distal profile slightly concave. Caudal fin with 10+9 principal rays, uppermost and lowermost rays unbranched, symmetrically forked, tip of lobes pointed (rounded); upper caudal-fin lobe length 32.0 (34.8) % of SL; median caudal fin length 14.9 (18.7) % of SL; lower caudal-fin lobe length 31.8 (34.9) % of SL. Lateral-line scales complete 24+1 (22+1). Pre-dorsal scales 10. Scale rows above lateral line 4½, below lateral line at pelvic fin base 3½, below lateral line at anal fin base 3½. Scale rows in transverse line on caudal peduncle ½2/1/2½. Total gill rakers ca. 12, rakers short, length of longest raker on first gill arch about half length of adjacent gill filaments. Vertebrae 16+13 (14+14).

***Coloration when fresh*** (Figs 3A, 4A). Head and body silvery-white, slightly cream to brownish dorsally, becoming lighter ventrally; indistinct longitudinal pinkish band on mid-lateral surface of body from below dorsal fin to caudal-fin base. All fins with semitranslucent interradial membrane and light cream to brownish rays.

***Coloration in alcohol*** (Figs 3B, 4B). Head and body pale yellowish brown; body slightly darker dorsally, becoming lighter ventrally. All fins semi-translucent.

#### Distribution.

Currently known from a subterranean creek in Cisodong 1 Cave, in the karst area at Klapanunggal, Bogor, West Java, Indonesia (Fig. 2). The creek drains into Cileungsi River, a tributary of the Bekasi River Drainage that is eventually emptied into the Jakarta Bay.

#### Etymology.

The specific epithet *klapanunggalensis* is derived from the type locality, the Klapanunggal karst area, which includes the Cisodong 1 Cave, Nambo Village. The name reflects the unique habitat and geological significance of the Klapanunggal karst area, where the species is likely endemic.

#### Comparisons.

The new stygobitic species *B.klapanunggalensis* can be distinguished from all congeneric species (including the epigean species *B.binotatus* allotopically co-ocurring in West Java), except the co-occurring subterranean species in Klapanunggal karst area B.cf.microps (see Kottelat et al. 1993: pl. 16) and a similarly subterranean Philippines species *B.pyrpholeos* (see Tan and Husana 2021: figs 1–7), in having a non-pigmented body (black dots, bars, stripes, blotches, and triangular markings all absent from body surface). The new species differs from *B.binotatus*, *B.microps* (including B.cf.microps), and *B.pyrpholeos* in lacking eyes (closed orbital concavity: Figs 3A, B, 4A, B; orbital rim absent: Figs 3A, 4B) [vs eyes present in *B.binotatus*, absent or present but usually small in *B.microps* (Weber and de Beaufort 1916; Kottelat et al. 1993: pl. 16; Haryono 2006: fig. 4A, B), and always present in *B.pyrpholeos* (Tan and Husana 2021: figs 1–7)]. In addition, *B.klapanunggalensis* sp. nov. can be distinguished from *B.binotatus* and *B.microps* by having a relatively short and rounded pelvic-fin axillary scale, its tip not reaching the posterior end of the pelvic fin base (Fig. 5A, B) (vs pelvic fin axillary scale slender, its tip distinctly beyond posterior end of pelvic fin base, in both of the latter; Fig. 5C, D)

Several morphometric characters of *B.klapanunggalensis* sp. nov. also differ from those of *B.binotatus*, *B.microps* (including B.cf.microps), and *B.pyrpholeos* (Fig. 6), including a long pectoral fin, its tip well beyond the pelvic-fin base origin (Figs 3, 4), 26.0–31.4% of SL [vs fin short, its tip not reaching origin of pelvic-fin base, 19.9–23.3 (mean 21.5) % of SL in *B.binotatus*, 20.2–23.8% in *B.microps*, and 18.6–22.6 (mean 20.9) % of SL in *B.pyrpholeos* (Tan and Husana 2021: figs 4–7, table 1]; a long pelvic fin, the depressed fin tip just reaching the anal-fin base origin (holotype) or well beyond (paratype) (Figs 3, 4) 21.5–24.4% of SL [vs fin short, tip usually not reaching anterior margin of anus, 17.2–20.5 (mean 19.0) % of SL in *B.binotatus*, 18.2–21.4% of SL in *B.microps* (tip just reaching anterior margin of anus in smallest specimen 48.6 mm SL), and 14.7–17.1% of SL in *B.pyrpholeos* (Tan and Husana 2021: figs 4–7, table 1]; relatively longer head 32.9–35.3% of SL [vs 25.3–29.7 (mean 27.3) % of SL in *B.binotatus*, 28.2–31.7% of SL in *B.microps*]; relatively greater pre-pectoral fin length, 32.6–33.6% of SL [vs 24.5–27.5 (mean 26.0) % of SL in *B.binotatus*, 26.0–30.2% of SL in *B.microps*]; and relatively greater pre-pelvic fin length, 54.0–59.6% of SL [vs 46.7–51.0 (mean 48.9) % of SL in *B.binotatus*, 48.6–54.9% of SL in *B.microps*]. In addition, *B.klapanunggalensis* sp. nov. also differs from *B.pyrpholeos* in having a greater anal-fin base length 9.7–11.8% of SL [vs 7.6–9.4 (mean 8.7) % of SL in the latter]; deeper caudal peduncle, 13.2–18.2% of SL [vs 11.6–12.9% of SL]; and dorsal, anal, and caudal fins with translucent interradial membranes and light cream to brownish rays (vs fins whitish anteriorly, orange posteriorly) (see Tan and Husana 2021: table 1, fig. 3, for *B.pyrpholeos*).

#### Habitat and ecology.

The specimens of *B.klapanunggalensis* sp. nov. were collected from two small adjacent pools in a vertical cave, drained by a seasonal subterranean tributary (Fig. 2D, E). Both pools, containing clear water and fine clay substrate, were situated 27 m downward from the cave entrance and fed by water percolating from the cave floor. The water depth of the pools when the specimens were collected was about 15 cm, approximately one-third of the pool depth (Fig. 2E). A flowstone above the pools, which conveyed percolating water to the latter, was characterized by microgour deposits, in which two crustacean individuals—*Stenasellusjavanicus* (Isopoda, Stenasellidae)—were observed. The fish in the pool remained stationary in still water, but began to actively swim when the water was disturbed.

#### Chronology of discovery.

The species was first observed in August 2020 by the second author (MIW)—together with teams from Latgab Caving Jabodetabeka, Indonesian Speleological Society (ISS), and Gema Balantara—in several small pools on the cave floor at two sites, each being on a different pitch (Fig. 2D). The first site comprised the two pools some 27 m depth from the entrance (see above), in which two barb individuals were observed. The second site was at the bottom of the cave (ca. 51.6 m depth), where more than 20 barb individuals were observed in pools with slow-flowing water. All of the barb individuals observed at that time lacked eyes and pigmentation. Individuals of the crustacean *Stenasellusjavanicus* were observed crawling on the bottom of the pools. Although the fish was captured in video format (Fig. 1A, B), specimens were not collected.

Later in July 2022, MIW and colleagues returned to the cave and collected two specimens from the same two pools as described for the first site in 2020, thus likely the same two individuals observed prior. Since access to the cave was technically difficult, only the first two pools were visited, with no attempts made to collect individuals from the deeper part of the cave (see Fig. 2D).

#### Notes on conservation status.

To date, *B.klapanunggalensis* sp. nov. has been found only in Cisodong 1 Cave (61 m in length; 51 m in depth). Although currently known from a single cave, it is possible that the species is also present in neighboring caves given the network of interconnected tributaries comprising the subterranean river system in the Klapanunggal karst area. This is supported by the presence of the crustacean *Stenasellusjavanicus* both in Cisodong 1 Cave and its type locality, Cikarae Cave, separated by about 6 km horizontal distance. Nevertheless, the overall distribution of the species is suspected to be likely limited to the Klapanunggal karst formation—an area of 66.64 km^2^, only 9.96% (6.64 km^2^) of which is under Indonesian Government protection (referred to as Kawasan Bentang Alam Karst Bogor, Zona Klapanunggal/No. 24 K/40/MEM/2020) —considering the troglomorphic features of the species indicating great adaptation to a unique habitat type (i.e., subterranean pools supplied by percolated water) within a relatively close system. While the subterranean tributaries within the karst ecosystem are likely interconnected, the entire Klapanunggal karst formation is relatively disjunct from other similar karst formations in Java, especially those in southern Central Java and Yogyakarta. With such a limited distribution, the potential for disturbance to the habitat and survival of this cave fish is considerable, particularly due to the type locality being outside the protected karst area (Fig. 2C). However, direct threats from locals to the existence of *B.klapanunggalensis* sp. nov. have not been identified, given that the cave location is far from settlements and difficult to access. Nevertheless, the Klapanunggal karst environment faces potential threats from extractive industries, particularly from limestone mining, which is prevalent in the region (Fig. 2A). Such activities have been known to impact karst ecosystems elsewhere and may pose risks to the habitat of *B.klapanunggalensis* sp. nov. if not managed sustainably.

*Barbodesklapanunggalensis* sp. nov. meets the criteria of a threatened species, such as restricted distribution, distinctive habitat, small population, and high potential threat level. By comparison, the congener *B.microps*, also recognized as a cave species, despite having the eyes not fully reduced compared to *B.klapanunggalensis* sp. nov., is Vulnerable in the IUCN Red List (Lumbantobing 2020), and as a “protected species” under Indonesian Government regulations. Clearly, further assessment of the status of *B.klapanunggalensis* sp. nov., based on IUCN Red List criteria, is necessary so that strategies for the conservation of the species and habitat can be formulated properly.

#### Remarks.

The two type specimens of *B.klapanunggalensis* sp. nov. showed a striking difference in overall body shape, the paratype appearing to be relatively more deep-bodied and plumper (body depth and width 43.1% and 22.5% SL, respectively) than the holotype. Although this suggested initially that the paratype was likely female (female cyprinid fishes commonly having a deeper wider body than males), dissection of the right side of the abdomen of the former revealed that the large and broadly expanded abdomen was due to the accumulation of viscous fluid, rather than the presence of gonad with eggs. Despite that in-situ observations in 2020 noted more than 20 individuals in two different locations, their male/female composition is still unknown.

*Barbodesmicrops* was originally described as *Barbusmicrops* by Günther (1868) on the basis of five specimens collected from Java Island, Indonesia. However, examination of the syntypes in this study confirmed that only three syntypes (BMNH 1845.4.22.334–336, 77.8–103.4 mm SL) conformed to *Barbodesmicrops*, the remaining two syntypes (BMNH 1845.4.22.341–342, 42.2–43.8 mm SL) representing a different, more slender-bodied cyprinid species.

In this study, *Barbodesmicrops* is regarded as a valid species. However, it was clearly stated in the original description of *Barbusmicrops* that the syntypes (likely referring to the former three syntypes mentioned above) were characterized by dark spots on the bases of the anterior dorsal and caudal fins, which aligns with the diagnostic characteristics of *Barbodesbinotatus*. Furthermore, the morphological features, including meristic and morphometric data of the three syntypes of *Barbusmicrops*, were consistent with those of non-type specimens of *Barbodesbinotatus* examined in this study. Therefore, *Barbodesmicrops* is likely to be conspecific with *Barbodesbinotatus* (see also Kottelat et al. 1993). A comparison between the type specimens of *Barbusmicrops* and those of *Barbusbinotatus* is necessary to strengthen this conclusion.

Aside from initially containing multiple species, the type material of *Barbusmicrops* also has unclear information about the exact locality as no additional geographical references—other than being collected in Java—was provided along the specimens. This, coupled with the ambiguous species diagnosis in the original description, has made any taxonomical reassessments of the group rather difficult, as the identity of *Barbodesmicrops* cannot easily be pinpointed to any single subterranean river system in Java; some of the systems—given their each being highly isolated and hydrologically disconnected from the others—may harbor their own hypogean barb species.

In fact, Weber and de Beaufort (1916) reported *Barbodesmicrops* (treated as *Puntiusmicrops*) based on non-type material collected from Gunung Sewu karst area in the central southern region of Java. It is, however, uncertain whether the specimens examined by the latter authors is really in the same subterranean system where the type specimens of *Barbusmicrops* were collected. Despite this uncertainty, we are certain that *B.klapanunggalensis* sp. nov. is not identical to these specimens from central Java not only because it is distinct morphologically, but also because the Klapanunggal karst area is hydrologically disconnected from all the other karst areas in Java, especially the one in Gunung Sewu karst area.

Another population also from the Klapanunggal karst area yet collected much earlier—herein referred to as Barbodescf.microps (MZB 60)—is also likely to be conspecific with *B.binotatus*. The specimens of MZB 60 were collected from a creek in Sileuwi Cave that has its own spring and continuously flows downstream to a larger tributary of the Bekasi River. The creek is also situated at a lower altitude than the type locality of *B.klapanunggalensis* sp. nov. (Cisodong 1 Cave), the latter of which contrastingly encompasses a pool-type habitat with no direct fluvial connectivity to the surrounding tributaries. In other words, the fluvial connectivity in the Sileuwi Cave to surrounding tributaries may allow gene flow between the epigean and hypogean populations of *B.binotatus*. In contrast, the hydrological isolation of the Cisodong 1 Cave is likely to maintain genetic isolation in *B.klapanunggalensis* as manifested in its distinct morphology.

The discovery of this new species of cave fish brings to six the total number of cave fish species endemic to Indonesia, the previously recorded species being: *Barbodesmicrops*, *Grammonusthielei* Nielsen & Cohen, 2004, *Bostrychusmicrophthalmus* Hoese & Kottelat, 2005, *Diancistrustyphlops* Nielsen, Schwarzhans & Hadiaty, 2009, and *Oxyeleotriscolasi* Pouyaud, Kadarusman & Hadiaty, 2012. Among the six species, only *Barbodesklapanunggalensis* sp. nov. and *Barbodesmicrops* have been recorded from Java (Rahmadi et al. 2018; Fig. 2B), the remaining species being known from various karst areas around Sulawesi Island and West Papua (Pouyaud et al. 2012).

## Supplementary Material

XML Treatment for Barbodes klapanunggalensis

## ﻿Additional information

### Author ORCIDs

Kunto Wibowo https://orcid.org/0000-0003-4465-3022

Anik Budhi Dharmayanthi https://orcid.org/0000-0003-4635-6099

Cahyo Rahmadi https://orcid.org/0000-0001-5928-9513

Daniel Natanael Lumbantobing https://orcid.org/0000-0002-1458-1470

### Data availability

All of the data that support the findings of this study are available in the main text.
